# Supplementary material for: Are carbon emissions trading and green financial instruments synergistic? -Comprehensive quantitative research based on content analysis
Source: PLoS One. 2024 Mar 7;19(3):e0298601. doi: 10.1371/journal.pone.0298601 (PMC10919601; doi:10.1371/journal.pone.0298601)
Supplement: S1 Data — (ZIP) [file pone.0298601.s001.zip › Data/地级市Z_F工作报告.zip!/╔╜╬≈/╜·│╟╩╨/└┤╫╘╣1⁄2╓┌║┼í╛┬φ┐╦╩2╛▌═°í┐_2019.docx]

各位代表：

　　现在，我代表市人民政府向大会报告工作，请予审议，并请市政协委员和其他列席人员提出意见。

　　一、2018年工作回顾

　　2018年是我市发展历程中极不平凡的一年。面对经济低位开局、环保倒逼、外部环境不确定性三大压力，全市上下坚持以习近平新时代中国特色社会主义思想为指导，全面贯彻党的十九大和十九届二中、三中全会精神，深入贯彻习近平总书记视察山西重要讲话精神，认真落实省委、省政府决策部署和市委七届四次、五次全会精神，以“先行区”“领跑者”“桥头堡”三大目标为引领，以“十项重点工作”为抓手，砥砺奋进，开拓创新，扭转了连续19个月经济下滑的困难局面，实现U型反转，推动新时代美丽晋城高质量转型发展取得良好开局。

　　2018年全市地区生产总值完成1351.9亿元，增长7.4%；一般公共预算收入完成125.3亿元，增长23.6%；规模以上工业增加值增长7.0%；全社会固定资产投资完成473.8亿元，增长10%；社会消费品零售总额完成452.8亿元，增长9.6%；城镇居民人均可支配收入完成32162元，增长6.7%；农村居民人均可支配收入完成13566元，增长8.4%；全市城镇新增就业和农村劳动力转移就业分别达到4.53万人和2.85万人；城镇登记失业率1.44%；居民消费价格涨幅2.2%。除空气质量二级以上优良天数外，其它约束性指标都较好完成了年度目标。

　　（一）产业转型迈出坚实步伐。我们把能源革命作为产业转型的重要基础，化解煤炭过剩产能240万吨，先进产能占比达66.2%，高于全省平均水平9.2个百分点。争创煤层气“一区三基地一中心”，全年煤层气产量达38亿立方米，煤层气产业总产值首次突破100亿元。把发展先进制造业作为产业转型的首要任务，设立“50亿+10亿”的光机电产业发展基金，出台“1+N”政策体系，着力推动富士康光机电产业核心技术、核心产业向晋城布局，富士康（晋城园区）全年总产值达到130亿元，增长40%；南村绿色智能产业园一期基本建成，入驻企业21家；晋钢智能科技产业园开工建设；在巴公园区谋划建设“造气岛”；中船重工新能源装备产业园和清慧制造轨道交通结构件项目进展顺利。制造业增加值占工业比重较上年提高0.3个百分点，煤炭产业占比下降0.5个百分点。把加快现代服务业发展作为产业转型的重要支撑，商贸物流、大数据、金融等重点行业发展迅速。服务业增加值占GDP的比重较上年提升0.9个百分点，增速高出GDP增速1.1个百分点。把全域旅游作为产业转型的重要突破口，“太行一号国家风景道”全线开工，晋城至郑州、焦作和长治以及市区到各县（市、区）的城际公交相继开通，王莽岭景区体制机制改革取得重大进展，太行古堡群世界文化遗产申报工作全面启动，华谊兄弟星剧场奠基开工，第三届海峡两岸神农炎帝文化旅游招商系列活动成功举办，在央视《魅力中国城》第二季竞演中摘取“十佳魅力城市”桂冠，全市旅游总收入和接待游客人次均增长25%以上。把项目建设作为产业转型的重要抓手，扎实推进转型项目建设年活动，省市重点工程完成投资232.6亿元，完成率106.3%，招商引资签约项目投资额和当年签约当年开工率超额完成省定目标。

　　（二）环保攻坚取得阶段性胜利。不断深化“环保不好，全局不保”的认识，把大气污染防治放在首要位置，建成了精准监控系统和指挥平台，完善了市、县、乡、村、企业五级网格化监管体系，出台了督查问责细则等规章制度，特别是聚焦“减煤”“治污”“管车”“降尘”“控烟”五个关键环节，科学施策、精准治理。启动市区建成区周边10公里范围内重污染企业的搬迁工作，对179家企业实施错峰生产；将禁煤区由41平方公里扩大到109平方公里；完成7.5万户清洁供暖改造任务，城区新增供热面积200万平方米；全天禁止重、中型货车在主城区通行，重污染天气实施机动车单双号限行；督促建筑工地严格落实“六个百分之百”，持续对市区40条主干道路和2条外环路实行“全覆盖、无缝隙、循环式”保洁和冲洗。环境空气质量综合指数同比下降11.6%，PM2.5和SO2两项指标均完成省定目标。严格落实“河长制”“河湖警长制”，水环境质量持续改善。土壤污染防治完成省定目标。环保督察整改基本到位。新增城市绿地面积63万平方米，完成国省营造林2.32万亩、中幼林抚育2.8万亩，生态环境持续改善。

　　（三）城市建设全面提速。启动《晋城市城市总体规划（2018-2035年）》修编，完成美丽晋城战略规划和海绵城市专项规划，城市设计试点工作被住建部作为典型经验向全国推广。老城更新与保护工程全面启动，一期房屋征收接近尾声，回迁安置前期工作基本完成，人居环境整治扎实推进。金村新区建设步伐加快，完成10平方公里核心区控制性规划和5平方公里城市设计，新开工5条骨干道路，6项公共服务设施项目完成前期。创新改造模式，新启动了一批城中村（棚户区）改造的前期工作。持续构建立体交通体系，太焦高铁顺利推进，晋城高速东口改造工程基本完工，阳蟒高速加快建设，晋阳高速八车道拓宽改造工程开工建设，晋城机场列入国家民用运输机场建设“十三五”中期调整规划，27条“三通”道路中18条建成通车，凤台街、泽州路和黄华街路面改造在一个月内高标准完成，体现了新时代的“晋城速度”。深入开展“双违”整治和“三洗六见一红旗”行动，国家卫生城市和国家森林城市通过复审，全国文明城市创建工作扎实推进。省级以上特色小镇总数居全省第一。

　　（四）乡村振兴战略深入实施。编制完成全市乡村振兴战略总体规划和“5+1”专项规划。出台《推进农林文旅康产业融合发展试点的实施意见（试行）》，统筹各类政策性资金5400万元，首批选取6个乡镇和12个典型村开展试点。成功申报省级“一县两片”有机旱作农业示范区。全市农村土地承包经营权确权登记颁证工作基本完成，农村集体产权制度改革进展顺利，泽州农地入市改革试点工作走在全国前列。启动农村人居环境整治三年行动计划，6个省级、20个市级美丽宜居示范村和2个集中连片示范区建设进展顺利。93个村入选第五批中国传统村落目录。建成“四好农村路”1303公里。陵川、沁水两县按期脱贫摘帽，在全省首批实现整市脱贫。

　　（五）改革创新亮点纷呈。重点领域改革取得突破。晋城开发区“一区四园”总体规划和起步区控制性详细规划编制完成，收储土地5000亩，基础设施和标准化厂房建设取得重大突破。高平开发区加快建设。沁水、阳城开发区顺利获批。对43户市属国有企业实施分类改革，推动市国有资本投资运营公司规范化运作，在全省率先成立市级新闻传媒集团，市公交公司改制重组挂牌运营，驻市央企、省企“三供一业”分离移交任务圆满完成。创新驱动持续发力。出台支持科技创新和“双创”的优惠政策，研发经费投入占GDP比重达到1.05%，排名全省第三。省级以上众创空间达到10家、“双创”平台达到15家、重点实验室和工程技术中心达到5个。新增国家级高新技术企业7家，总数达到49家。民营经济活力增强。启动实施“236”行动和“小升规”计划，兑现奖励2754万元。建立小微企业信用贷款风险互助补偿机制，帮助188家小微企业获得授信1.05亿元。新登记市场主体25597户，同比增长12%。我市被列为全省民营经济改革发展示范市。

　　（六）民生社会事业全面进步。全年民生支出达187.4亿元，占一般公共预算支出的82.1%。率先在全省实现农村寄宿制学校后勤保障全覆盖。市区组建11个办学集团，义务教育集团化办学全面启动。学前教育城乡一体化省级试点稳步推进。凤城中学高中部、综合实践基地基本完工。县乡医疗卫生机构一体化改革扎实推进，基本公共卫生考核全省第一。率先在全省完成城乡居民基本医疗保险制度整合，率先在全省实现全国异地就医费用即时结算。新建60个农村老年人日间照料中心。新开工棚改住房6873套，基本建成2330套。新（改）建人行天桥4座。新（改）建公厕125座，完成农村旱厕改造3088户。全面启动国家公共文化服务体系示范区创建工作，上党梆子《太行娘亲》入选国家舞台艺术精品工程重点扶持剧目，电视剧《一代名相陈廷敬》在央视热播。安全生产创历史最好水平。扫黑除恶专项斗争扎实推进。十件民生实事全部落实。群众安全感和执法满意度实现全省“七连冠”。荣膺全国双拥模范城“三连冠”。荣获全国“质量魅力城市”、创建“平安医院”先进市、“全国无偿献血先进市”等多项荣誉。入选“中国地级市民生发展100强”。

　　（七）政府自身建设得到加强。严格落实政府系统全面从严治党主体责任，着力加强“三基建设”，深入推进党风廉政建设和反腐败斗争。坚持“晋城的事，大家想、大家说、大家干”，进一步激发了政府系统党员干部干事创业的积极性和创造性。有序推进政府机构改革。积极配合市人大常委会制定《晋城市文明行为促进条例》《晋城市太行古堡群保护条例》《晋城市大气污染防治条例》，严格执行市人大及其常委会的决议决定，主动接受人大法律监督和工作监督、政协民主监督，办理人大代表建议495件、政协提案515件。依法办理行政复议案件109件，市长热线办复率99%以上。深化“放管服效”改革，高平市相对集中行政许可权改革省级试点推进顺利；备案类项目全面实行承诺制；动态调整了18个部门的117项权责清单事项；行政审批事项前置申请材料精简41%；新建成的市级政务服务中心全面启用，审批事项入驻率达到98%；“3545”专项改革均提前完成省定目标任务，不动产登记工作走在全国前列。“13710”督办制度深化拓展，政府效能不断提升。

　　各位代表，过去一年的成绩来之不易。这是习近平新时代中国特色社会主义思想科学指引的结果，是省委、省政府和市委科学决策、正确领导的结果，是市人大、市政协监督支持、积极参与的结果，是全市人民同心同向、奋力拼搏的结果。在此，我代表市人民政府，向全市人民，向各民主党派、工商联和无党派人士，向各位人大代表、政协委员，向驻市部队指战员、武警官兵、公安干警，向各位老领导、老同志，向所有关心支持晋城改革发展的各界朋友，表示崇高的敬意和衷心的感谢！

　　在肯定成绩的同时，我们也清醒地认识到，我市发展仍面临诸多困难和挑战，政府工作还存在很多不足。主要表现在：产业转型升级步伐不快，经济稳步增长、持续向好的基础还不牢固；招商引资力度小，大项目、好项目、转型项目少；民营经济发展滞后，活力仍然不足；污染防治压力较大，生态环境保护任重道远；民生事业还存在不少短板；政府职能转变还不到位，营商环境有待进一步优化；一些干部适应新时代发展要求的能力不足，抓落实的本领亟待提高；少数干部懒政怠政，一些领域不正之风和腐败问题不容忽视。认真审视，最大的问题是改革开放不够，根本的原因是思想解放不够。要彻底解决这些问题，出路在改革开放，关键在解放思想。对此，我们必须坚定信心、迎难而上，以更大的决心和更有效的举措，坚决根除困扰我们的陈旧观念，坚决冲破阻碍发展的各种羁绊。

　　二、2019年工作安排

　　2019年是中华人民共和国成立70周年，是全面建成小康社会的关键之年，是在“两转”基础上全面拓展新局面的攻坚之年，也是推动新时代美丽晋城高质量转型发展取得新成果的重要一年。今年政府工作的总体要求是：以习近平新时代中国特色社会主义思想为指导，全面贯彻党的十九大和十九届二中、三中全会精神，统筹推进“五位一体”总体布局，协调推进“四个全面”战略布局，深入贯彻习近平总书记视察山西重要讲话精神，认真落实中央及省委省政府重大决策部署，在市委坚强领导下，坚持稳中求进工作总基调，坚持新发展理念，坚持把供改和综改相结合作为经济工作主线，贯彻“巩固、增强、提升、畅通”八字方针，进一步稳就业、稳金融、稳外贸、稳外资、稳投资、稳预期，扎实开展“改革创新、奋发有为”大讨论，按照“主攻三大目标，打赢三大攻坚，补齐三大短板，干好三件大事，守住三条底线”的要求，解放思想、凝聚共识、先行先试，加快推动新时代美丽晋城高质量转型发展取得新成果，为全面建成小康社会收官打下决定性基础，以优异成绩迎接中华人民共和国成立70周年。

　　主要预期目标是：地区生产总值增长6.5%左右，全社会固定资产投资增长7%左右，社会消费品零售总额增长7.5%，一般公共预算收入增长7%以上，城乡居民人均可支配收入分别增长6.5%和6.5%以上，居民消费价格涨幅控制在3%左右，城镇新增就业4.53万人，城镇调查失业率、城镇登记失业率分别控制在6.5%和4.2%以内。各项约束性指标控制在省下达指标以内。

　　2019年指标的设定，是立足决胜全面建成小康社会、把握我市转型发展阶段性特征和经济运行趋势而确定的，突出了高质量发展要求，体现了稳中求进总基调，彰显了“山西大发展、晋城要先行”的决心。

　　实现上述目标，任务繁重艰巨，必须扭住“十项重点工作”不放松，持续发力、久久为功，一件一件抓落实，一项一项见成效。

　　（一）扭住工业转型不放松，加快构建现代产业新体系。产业转型是晋城高质量发展的内在要求，工业转型是晋城产业转型的重中之重。我们要坚持把工业作为驱动全市产业转型的主引擎，紧扣“3+5”政策体系，一手抓传统产业改造升级，一手抓新兴产业培育壮大，不断优化产业结构，提升产业层次。

　　持续深化能源革命。抢抓国家支持我省开展能源革命综合试点的战略机遇，积极争创全国煤层气“一区三基地一中心”，筹划建设一批煤层气勘探开发项目以及输气管网和储气设施，力争今年将产能增加到70亿立方米，5年内增加到200亿立方米。继续运用市场化法治化手段退出煤炭过剩产能，提升先进产能占比，稳妥处置已关闭退出煤矿的资产债务问题。加大风能、太阳能、生物质能、地热能等新能源开发和利用力度，重点抓好陵川中电投、沁水国电投、沁水远景汇合等风电项目和兰能玉溪、金驹胡底等瓦斯发电项目。

　　着力打造全省先进制造业基地。以高端化、智能化、绿色化、服务化为方向，不断提升制造业整体素质。光机电，以富士康为核心，紧盯上下游企业，着力培育光机电一体化的产业集聚区，打造全省全产业链发展的样板。重点推动富士康投资63亿元的光机电项目和首批招商的20个项目尽快落地。煤机装备，重点抓好金鼎煤机、江淮重工、天巨重工等企业，发展成套煤机装备制造。新能源装备，重点加快中船重工新能源装备产业园二期和长征动力三元锂电池项目一期建设。轨道交通装备，重点推动清慧年产5000万件轨道交通新型材料结构件项目一期投产。冶铸业，加快推进南村绿色智能铸造创新产业园二期和晋钢集团智能铸造科技产业园一期工程。煤化工，重点开工建设巴公园区“造气岛”。同时，着力推动陶瓷、丝麻纺织和现代医药等产业上档升级。用好工业发展专项资金，支持工业企业加快转型。

　　推动制造业和服务业深度融合。以商贸物流、大数据、金融服务等生产性服务业为重点，多措并举，加快发展，不断提升服务制造业的能力和水平，着力推动制造业与服务业深度融合，互促共进。商贸物流业，紧密结合我市正在构建的立体化交通体系，按照打造中东部地区现代物流节点城市的新定位，谋划一批物流项目。重点抓好兰花、蓝远、义乌国际商贸城等物流园区建设和苗匠物流园区招商运营工作。大数据产业，以打造中等智能城市全国样板为目标，深化与阿里巴巴集团的合作，加快实施“1183”工程，实现全市大数据应用重大突破。金融服务业，以创建国家级金融支持资源型经济转型发展改革创新试验区为契机，提升企业直接融资比重，健全完善小微企业信用贷款风险互助补偿机制，推动政府性融资担保机构增资展业，积极防范化解金融风险，持续引导金融机构支持实体经济发展。扎实做好国家标准化工作综合改革试点相关工作，以先进标准助力产品质量提升、产业转型升级。

　　坚持创新驱动发展。把产业转型的基点放在创新上，支持重点企业建设省级以上重点实验室和工程技术中心。推进产学研深度融合，重点推动山西大学与富士康共建晋城纳米光机电产业研究中心和光机电研究生培训基地。实施高新技术企业五年倍增计划，今年力争新增国家级高新技术企业3家。坚持“不求所有、但求所用”的理念，进一步完善招才引智政策体系。支持企业建设“千人计划”专家和博士工作站，引进培养高技能人才。发挥职业教育集团作用，推进新型学徒制，培养本土实用型人才。对于各类所有制企业引进的优秀人才，只要符合政策，政府都将给予住房、子女教育等方面的支持服务，为加快产业转型提供人才保障。

　　（二）扭住大景区整合不放松，全面展现太行旅游名城新魅力。晋城既有大自然恩赐的壮美山水风光，也有老祖宗创造的不朽文化遗产，更有革命先辈留下的宝贵红色基因。我们一定要挖掘好、整合好、利用好这些得天独厚的资源优势，把晋城建成国内一流、国际知名的新一线旅游城市。

　　加快大景区整合步伐。以王莽岭开发为龙头积极与省文旅集团等国内外大型旅游企业对接合作，按照5A级景区标准，全面启动景区基础设施提升和游客中心、锡崖沟水镇等项目建设，确保在2020年大交通体系形成时具备相应的游客接待能力。组建晋城市文旅投融资平台。有重点地推进太行古堡密集区资源整合和开发利用，做好太行古堡申遗工作。选取50处左右古民居开展民间认领保护活动，提升文物古建“活化利用”水平。按照“一县一大景区”的发展思路，加快推动皇城相府、珏山、太行洪谷、炎帝陵等其它大景区建设。

　　完善旅游基础设施。续建“太行一号国家风景道”244公里，年内完成投资15亿元。合理布局、加快建设集散中心、旅游厕所、休闲驿站、房车营地等公共设施，着力构筑环太行山旅游景区循环圈。继续解决主干道至各景区“最后一公里”问题。

　　提升旅游服务品质。提升餐饮住宿、休闲娱乐等传统服务业发展水平，引导支持民宿客栈规范发展，探索开发避暑康养、文化研学、低空体验、户外体育等新业态，打造复合型旅游目的地。加快华谊兄弟星剧场项目建设，改进《再回相府》和司徒小镇花车巡游演艺模式，进一步放大优质项目品牌效应。加强全域旅游标准化建设，年内新增5个3A级以上景区。持续开展旅游从业人员素质提升工程，加强文旅市场综合监管，进一步整顿和规范旅游市场秩序。加强智慧旅游建设。在中央电视台投放城市旅游形象宣传片。以海峡两岸神农炎帝文化旅游招商系列活动为龙头，统筹办好太行山国际文化旅游节、“体验魅力中国城”等重大活动，持续做好非遗、演艺进景区工作，叫响叫亮“晋善·晋美·晋城”品牌。

　　（三）扭住农林文旅康产业融合发展不放松，奋力谱写乡村振兴新篇章。实施乡村振兴战略是新时代“三农”工作的总抓手，农林文旅康产业融合发展整市推进试点是省委、省政府赋予我们的重要使命。我们要勇于担当、不辱使命，积极探索、先行先试，以此带动农业增效、农民增收，实现乡村振兴。

　　加快推进农林文旅康产业融合发展。依托优势资源，突出融合发展，把农林文旅康产业融合发展与全域旅游结合起来，一同推进。探索设立农林文旅康产业融合发展基金，下大力气推动现有试点在产业发展、要素保障、体制机制创新上取得突破，在此基础上，再把一些基础好的乡镇、村纳入试点推进。每个县（市、区）都要找准突破口重点探索，年内推出一批先进典型。

　　持续深化农村人居环境整治。学习浙江“千村示范、万村整治”经验做法，持续抓好“五大专项行动”。扎实推进泽州县省级农村人居环境整治示范县和14个省级示范村建设，加快形成能复制、易推广的建设、运行、管护机制。新（改）建“四好农村路”990公里。继续抓好“大棚房”问题专项清理整治行动，毫不松懈抓好非洲猪瘟疫情防控工作。

　　大力发展特色现代农业。实施好粮食产能工程。积极推广功能农业、有机旱作农业，完成特色产业提质增效1万亩,杂粮种植面积稳定在60万亩以上。持续推进“三品一标”认证和区域农产品公用品牌建设，培塑3个以上功能农业品牌。大力发展“互联网+现代农业”。大力培育农业产业化龙头企业，力争亿元以上龙头企业达到13家。

　　全面深化农村改革。完善农村承包地“三权分置”制度。扎实推进农村集体产权制度改革，有序开展经营性资产股份制改造。深入推进农村“三块地”改革试点，探索农村“三变”改革路径。着力培育家庭农场、农民合作社等新型经营主体，支持各类人才返乡下乡创业创新，统筹推进农村各项改革，进一步激发农业农村发展活力。

　　继续打好脱贫攻坚战。坚持摘帽不摘责任、不摘政策、不摘帮扶、不摘监管，紧盯“两不愁三保障”目标，持续抓好产业扶贫、特定贫困群体保障、政策措施落实、搬迁后续发展、驻村帮扶五项重点工作，巩固脱贫成果，提升发展能力。建立返贫预警机制，减少和防止脱贫人口返贫。

　　（四）扭住“最后一公里”政策落地不放松，再创民营经济新辉煌。晋城的民营经济曾经创造过“三分天下有其二”的辉煌，在新一轮大发展中，只能壮大、不能弱化。我们要坚持“一把尺、一样亲、一家人”的理念，以建设全省民营经济发展改革示范市为抓手，全力推动我市民营经济发展步入快车道、再上新台阶。

　　狠抓政策落实。主动对标省委、省政府支持民营经济发展“30条”，认真落实好市委、市政府出台的决定和具体措施。严格落实减税降费各项优惠政策。实施市场准入负面清单制度，着力消除民营企业在准入许可、经营运行、招投标等方面的不公平待遇。依法保护企业家人身和财产安全，抓好清理政府部门和大型国有企业拖欠民营企业账款工作。完善领导干部联系民营企业制度，健全企业家参与涉企政策制定机制，着力构建“亲”“清”新型政商关系。

　　继续推进“236”行动和“小升规”企业培育工作。以打造“双创”升级版为抓手，持续加大“双创”示范基地和省级以上众创空间建设力度，着力推动中小企业走“专精特新”道路。建立问题台账和沟通协调机制，抓好一批民营企业转型项目。重点培育“小升规”企业30家，继续抓好一批服务业“小上限”企业。

　　启动实施企业家队伍素质提升“421”计划。年内培训领军企业家40名，高级经营管理人员200名和中小微企业经营人才1000名。加强与德国国际合作机构（GIZ）合作，重点培训100名企业家。

　　（五）扭住“深化转型项目建设年”活动不放松，着力推动招商引资实现新突破。今天的项目质量决定明天的发展质量，今天的招商导向决定明天的发展走向。当前，我市产业发展的方向已经明确，项目落地的基础条件已经具备。我们要坚持把项目建设和招商引资作为“一把手工程”，精准发力，强势推进。

　　全力推进转型项目建设。按照省委、省政府和市委要求部署，高标准谋划实施一批打基础、利长远、补短板、增动能的新项目、大项目、好项目。加快推进320个亿元以上项目建设，年内完成投资496亿元，重点抓好总投资为1073.4亿元的60项省市重点工程，年内完成投资208.9亿元。积极争取市县政府新增债券额度，申报国家和省技术改造专项资金，引导社会资本投资重大项目建设。进一步改进重点项目清单管理、手续集中办理、项目集中开工、“一月一调度、双月一例会、半年一点评、年终一总结”“七位一体”抓督查等做法，对亿元以上重大转型项目探索实行领办和帮扶服务，加快项目落地见效。严厉打击阻工扰工、围标串标等违法行为，坚决整治“吃拿卡要”、不作为、乱作为等影响重点工程建设的问题，大力营造服务支持转型项目的浓厚氛围。

　　全面提升招商引资质量和效益。坚持“系统定向精准”的理念，突出产业链招商、专业化招商、社会化招商，切实提高招商引资的针对性和实效性。以主导产业为招商重点，紧扣全省招商图谱和我市招商地图，在建链、补链、强链上取得突破。改革招商队伍管理体制和薪酬制度，优化设置产业招商小分队，打造专业化招商队伍。持续实施“晋商晋才回乡创业创新”工程，精心组织好女企业家高峰论坛等活动，积极探索以商招商、委托招商、乡情亲情同学情招商等多种方式，调动各类资源，扩大招商成效。充分发挥开发区招商引资主战场、主平台作用，晋城经济技术开发区至少引进落地5个总投资10亿元以上的转型项目，其它开发区至少引进落地2个总投资5亿元以上的转型项目。在开发区之外，各县（市、区）至少要再引进落地3个总投资10亿元以上的转型项目。

　　（六）扭住城市建设管理不放松，彰显具有深厚文化底蕴和现代特色风貌的城市新形象。经过一年多的探索与实践，城市建设的思路已经明确，规划基本确定，一批重大项目正在实施。我们要进一步加大建设力度、加快工程进度、提升管理水平，着力在统筹上下功夫、重点上求突破、特色上做文章，全面提升城市品质。

　　扎实推进老城更新与保护。按照“提升、保护、延续、注新”的规划理念，用3-5年时间，投资72亿元，采用微改造模式，突出地域特色，打造底蕴深厚的历史文化街区和自然优美的景观街区。今年，重点完成东西大街、景德路、景忠路等主要路网改造以及南大街主要建筑的立面改造；启动实施景德桥公园、景忠桥公园、东滩会、西滩会景观改造和东河、西河景观整治，加快老城居民回迁楼建设，明年底全部建成；统筹推进文庙巷、青龙巷和南谷洞等历史文化街巷提升改造，谋划启动东西大街以北区域改造的前期工作。

　　加快金村新区建设。按照县级实施、市级统筹的模式，整体推进金村新区建设，力争3-5年内投资150亿元，形成城市的另一个增长极。今年，重点是开工建设高铁东站站前广场，实施周边可视山体绿化，按照快速公交的理念启动丹河快线“1+3”一期工程，确保2020年底高铁通车时，站前广场、公园、“两纵四横”六条站前道路、公交和旅游换乘中心以及与市区连接道路等配套工程全部完工。

　　有序推进城中村（棚户区）改造。坚持拆迁与建设并举、安置与开发并重，积极引进资金实力雄厚的大品牌开发商，重点选择一批前期工作扎实的村（社区）开工建设，并切实加大争取国家棚改专项债券力度。制定退出机制，对批复后三年以上仍未开工或开工后进展缓慢的城中村（棚户区）改造项目，推动开发商有序退出。

　　加强城市基础设施建设。重点抓好总投资64亿元的26项“三通”工程，确保畅安路北延、景西南路南延、金匠街西延等21条道路年内通车或具备通车条件。扎实推进中原街改造及综合管廊建设等PPP项目。持续开展城市“双修”，重点对文博路、红星街等6条道路和22条小街巷实施改造和修补。加快实施总投资约34亿元的新“三源”工程，完成张峰水库引水主体工程，开工建设第三水厂项目；开工建设阳城电厂至市区集中供热管网工程；完成新气源项目长输管道、分输站及附属设施建设。加快城市主要路口立体交通设施建设，新（改）建一批公共停车场，实施老旧小区电梯等配套设施改造补贴工程。

　　提升精细化管理水平。加快建设全市统一的大数据云平台和政务服务平台，夯实智慧城市基础。深化城市管理执法体制改革，进一步推动重心下移，提高执法效能。加大“双违”整治力度，重点查处“边整治边违建”的问题。以创建全国文明城市为统领，持续巩固“三洗六见一红旗”行动成果，不断提升城市品质。

　　积极推动区域协调发展。扎实推进行政区划调整工作，解决“一市一区”问题。统筹推进县域经济特色化、差异化发展。扎实推进润城、巴公、神农3个国家级特色小镇和大阳、北留、端氏、嘉峰4个省级特色小镇建设。积极推进农业转移人口市民化。推动交通、信息、能源等基础设施向农村延伸，促进城乡基本公共服务均等化。

　　（七）扭住重点领域改革不放松，激发转型发展新活力。发展前进一步，就需要改革前进一步。要牢固树立“允许改革有失误，但不允许不改革”的鲜明导向，坚持“供改”和“综改”相结合，“补考”和“赶考”一起抓，持续深化重点领域改革，努力跻身全省改革第一方阵，以改革“一子落”带动转型“满盘活”。

　　深化“放管服效”改革。在全省率先全面推行相对集中行政许可权试点改革，市县两级全部组建行政审批服务管理局，实行“一个部门、一颗印章”管审批。引深企业投资项目承诺制改革，在实施“承诺制+并联审批”“承诺制+标准地”“容缺受理”等方面走在全省前列。巩固深化“3545”专项改革，继续压缩企业开办、不动产登记、工程建设项目审批时间。全面推开“证照分离”“多证合一”改革，推进“照后减证”，降低准营门槛。全面推进“双随机、一公开”监管。深化公共资源交易领域市场化改革。

　　深化国资国企改革。启动第二批14户市属国有企业改革工作，年底实现市属国有企业深化改革全覆盖。围绕“四个平台”，推动国有资本投资运营公司做好资本运作、股权管理等工作。加快推动兰花集团转型、天泽集团上市。适时整合公益类国有企业，探索设立公益类国有资本投资运营公司。推进国有企业混合所有制改革，推动商业类国有资本向优势领域集聚，向专业化方向重组。基本完成国有企业剥离办社会职能任务。

　　加快开发区改革创新发展。推动各开发区积极复制转型综改示范区经验，在基础设施建设、“三化三制”改革、招商引资、优化营商环境等四个方面取得明显成效。积极推进陵川省级开发区申报工作。鼓励开发区与发达地区或优势企业合作共建产业园区。坚持“以亩均论英雄”，加强各开发区投资强度、产出强度、税收强度考核。支持晋城经济技术开发区大胆创新、先行先试，进一步发挥国家级开发区的示范引领作用。

　　与此同时，按照市委改革清单，统筹抓好金融、财税、科技等各领域改革工作。

　　（八）扭住融入中原不放松，全力构建开放型经济新格局。晋城是山西对外开放的重要门户，也是中原城市群核心发展区城市。我们一定要深化“晋城不是边缘城市，而是开放前沿”的认识，加强区域合作，扩大对外交流，在更大范围、更宽领域、更高层次上推进新一轮对外开放。

　　深度融入中原。发挥优势、突出特色，深化与中原城市群其它城市在交通基础设施互联互通、文化旅游、商贸物流、装备制造、能源供给等方面的合作。同时，加强与京津冀地区协作联动发展，对接长三角一体化和粤港澳大湾区，深度融入“一带一路”战略。

　　构建开放通道。铁路方面，加快太焦高铁晋城段建设，协调做好呼南高铁、晋城—运城客专前期工作，开工建设5条“公转铁”专用线，推动嘉南铁路恢复开工。公路方面，加快推进国道342改线工程，推动国道207线新房洼至省界瓶颈路段改造工程年内完工，力争新国道207线陵川—长治一级公路改造工程、省道阳济线重载交通示范改造工程年内开工；建成阳蟒高速，推动晋阳高速八车道拓宽改造工程全线开工，加快东南环高速、西南环高速和阳城—运城高速、沁水—安泽高速前期工作，协调推进陵川王莽岭—新乡、阳城蟒河—济源两条出省高速公路建设。机场方面，做好晋城民用机场前期工作，同步谋划空港新区，启动机场道路建设和村庄搬迁工作；加快推进阳城通用机场建设。

　　搭建开放平台。继续做好中国（山西）自贸试验区晋城片区的申报设立工作。促进兰花保税物流中心与河南保税物流中心全方位合作，加快跨境电商平台和晋城铁路口岸建设。启动实施对外贸易主体培育三年行动计划，发挥好海关、保税区等开放平台作用，支持外贸企业做大做强，支持特色产品、优势产品扩大出口。做好外事、侨务、港澳、对台工作，搭建具有晋城特色和世界影响力的交流平台。

　　（九）扭住环保攻坚不放松，厚植“生态立市”新优势。生态是晋城的优势、晋城的品牌，也是晋城永续发展的巨大财富。我们要充分认识到“保护环境就是保护生产力，改善生态就是发展生产力”，坚定不移打好污染防治攻坚战，牢牢守住环境质量底线和生态保护红线，真正在解决突出环境问题中倒逼产业转型，在守护绿水青山中收获金山银山。

　　以更精准的举措打赢蓝天保卫战。持续优化产业结构，重点加快市区周边10公里范围内冶铸、煤化工企业退城入园步伐。持续优化能源结构，优先发展清洁化燃煤供暖和工业余热供暖，建立以集中供热为主，煤改气、煤改电为辅，3#优质煤兜底的清洁供暖体系，推动清洁取暖和散煤替代向农村扩展。持续优化运输结构，推进大宗货物及中长距离货物运输“公转铁”，强化非道路移动机械、混凝土搅拌车标准化管理，严格油品管控，持续打击黑油窝点。严格落实“六个百分之百”要求，全面推进建筑工地绿色施工，加大高速公路以及市区道路的常态化清扫、冲洗力度。严格落实错峰生产、错峰运输和重污染天气应急减排措施。加快推进中石化晋城油库搬迁。进一步压紧压实网格化环境监管责任，下大力气提高环保科学化水平。

　　以更大的力度打好碧水保卫战。统筹推进“五水同治”，强力推进丹、沁两河水环境综合整治。加大重点河流流域化工企业管控力度，实行排放日总量控制，重点抓好晋煤天溪、华昱煤化工等一批重点企业废水零排放改造工程。加快城镇污水管网向城郊村延伸覆盖，统一规划建设重点乡镇污水处理设施。巩固黑臭水体综合整治成果，扎实推进市区“清水复流”工程。

　　以更实的工作推进净土保卫战。全面完成土壤污染状况详查。编制完成一般工业固废和危险废物处置设施规划，重点加大对全市煤矸石、粉煤灰的综合治理力度，统筹建设循环高效的工业固体废物综合处置利用项目。扎实开展农业面源污染防治和农田残膜治理试点工作，持续推进秸秆禁烧和综合利用。

　　以更高的标准提升生态环境建设水平。严守生态保护红线，完成国省营造林2.21万亩、中幼林抚育0.65万亩。持续推进环城生态圈和城市绿道建设。完成文昌、春晓等5个游园工程，开工建设时家岭公园和凤台公园，持续抓好新建城市道路绿化美化。持续推进国家级低碳试点市和循环经济示范市建设。

　　各位代表，在2018年环保最紧要的关头，全市上下众志成城、共克时艰，凭着壮士断腕的决心和意志，凭着血战到底的勇气和努力，取得了大气污染防治阶段性胜利。在此，我代表市人民政府向全市人民致以崇高的敬意和衷心的感谢！

　　（十）扭住群众关心的热点难点问题不放松，推动各项民生社会事业迈上新台阶。人民对美好生活的向往是我们始终不渝的奋斗目标。我们要牢固树立过紧日子的思想，严控“三公”经费预算，按照不低于5%的比例压减一般性支出，继续把新增财力优先用于保障和改善民生,着力解决好人民群众最关心最直接最现实的利益问题。

　　千方百计扩大就业。扎实做好高校毕业生、去产能安置职工、农村劳动力、退役军人、就业困难人员等重点群体就业工作，确保全市零就业家庭至少一人稳定就业。加强全方位公共就业服务，提高劳动者就业能力。

　　办好人民满意教育。以率先在全省实现教育现代化为目标，构建城乡一体、优质均衡、设施先进、协调发展的现代教育体系。深入推进义务教育一体化改革，调整优化学前教育布局，促进职业教育产教融合，办好特殊教育，支持规范民办教育发展。强化教师队伍建设。新（改）建13所幼儿园。推动凤城中学高中部、综合实践基地尽快投入使用，太原科技大学晋城校区学生宿舍楼（二期）年内建成并投入使用，东南新区学校开工建设，完成晋城中专整体搬迁。在南岭爱物学校筹建晋城一中分校。

　　加快健康晋城建设。深化县乡医疗卫生机构一体化改革、城市医联体建设和公立医院改革，加快医保支付方式、公立医院医药价格和药械采购改革，促进“三医联动”。加大疾病防控和食品药品监管力度。加大中西医人才引进培养力度。深入开展全民健身运动。积极开展全国居家和社区养老服务改革试点工作。完成荣军康复医院整体迁建工程，推动市人民医院易址扩建项目上半年开工建设、省中医院与瑜园老年公寓项目深度合作。力争年内实现创建国家卫生县城全覆盖。

　　完善住房保障体系。实施好总投资34亿元的保障性安居工程，建设安置房4103套。大力推广装配式建筑，加快推进住宅全装修工作。建立健全房地产市场调控长效机制，促进房地产市场平稳健康发展。扩大住房公积金制度覆盖面，提升管理服务水平。

　　提升文化服务水平。培育和践行社会主义核心价值观，深入实施公民道德建设工程。全力创建国家公共文化服务体系示范区。深入推进乡村文化记忆工程和文化惠民工程。推动优秀传统文化、非物质文化遗产传承发展。承办好第二届全国青年运动会有关项目。市民广场全部完工，文化艺术中心完成部分主体工程。

　　强化民生兜底保障。落实城乡居民基本养老保险待遇确定和基础养老金正常调整机制，稳步提高各项社会保险待遇水平。继续提高城乡低保和特困人员救助供养标准。大力发展妇女、儿童、残疾人、慈善和红十字等事业。

　　维护社会安全稳定。全面贯彻总体国家安全观，紧盯重大敏感节点，提高风险预知预警预判和应对处置能力。完善社会矛盾纠纷多元化解机制，做实做细各类利益诉求群体的信访维稳工作。做好民族宗教工作和援疆工作。支持国防建设，加强双拥和退役军人服务管理工作。更好发挥工会、共青团、妇联等群团组织作用，完善基层群众自治制度，推动社会治理重心向基层下移。加强气象、地震、人防、治超等工作。持之以恒抓好安全生产，坚决杜绝重特大安全事故。完善应急管理工作机制，切实提升防灾减灾救灾能力。加快推进“雪亮工程”建设，持续推动扫黑除恶专项斗争向纵深发展，全力维护社会大局和谐稳定。

　　今年大事多、要事多，防风险、保平安的任务更加艰巨，我们一定要坚持底线思维，增强忧患意识，坚决有力防范化解各类重大风险，牢牢守住社会安全稳定这一底线。

　　各位代表，在办好省政府确定的8件民生实事的基础上，今年市政府将再为全市人民办好8件民生实事。(1)实施行政村4G网络全覆盖工程；(2)实现全市80周岁以上老年人高龄补贴全覆盖；(3)为全市约2.2万名新生儿进行多种遗传代谢病串联质谱和耳聋基因分析检测及诊断；(4)实施农村饮水安全巩固提升工程，受益人口9.83万人；(5)在主城区新建10座人行天桥；(6)新（改）建68座公共厕所；(7)继续实施清洁供暖改造工程，年内完成4.62万户改造任务；(8)实施市域公交一体化提质工程，新增、优化公交线路，购置110台新能源纯电动公交车，对124个公交站牌进行智能化改造。

　　我们将信守承诺，坚决把好事办好、实事办实，让广大群众有实实在在的获得感、幸福感、安全感。

　　三、全面加强政府自身建设

　　在习近平总书记视察山西重要讲话精神指导下，我省在“两转”基础上拓展了党的建设和党的事业新局面，在“晋城的事大家想、大家说、大家干”的浓厚氛围中，我市实现了新时代美丽晋城高质量转型发展的良好开局。我们要以“改革创新、奋发有为”大讨论为契机，乘势而上，对标一流，开拓前行，着力打造人民满意的政府。

　　加强政治建设。坚持以习近平新时代中国特色社会主义思想为指导，树牢“四个意识”，坚定“四个自信”，坚决做到“两个维护”。自觉扛起全面从严治党主体责任，切实加强政府系统党的建设。扎实开展“不忘初心、牢记使命”主题教育，持续加强“三基建设”，创造性地贯彻落实党中央、国务院，省委、省政府和市委各项决策部署。

　　提高施政能力。全面加强对政府工作人员的教育培训，继续推动各级干部走出去，远学江浙、近学四邻，开拓视野、增长才干。加强政策研究和经济运行监测，开展好第四次经济普查，推进智库建设。大力践行清单工作法、一线工作法、群众工作法，切实增强推动高质量发展的能力。

　　严格依法行政。自觉运用法治思维和法治方式推进工作，更好地带动全社会尊法守法。严格执行市人大及其常委会的决议决定，认真办理人大代表建议、政协提案。加强政府立法工作，完善政府法律顾问制度。推进综合行政执法体制改革，严格规范公正文明执法。继续开展“向人民承诺”活动，全面推进政务公开和政府信息公开，自觉接受人大、政协监督以及社会、舆论监督，强化审计监督。

　　优化政务服务。牢固树立“人人代表政府形象”“事事体现营商环境”的理念，按照“三对”“六最”的要求，最大限度提升企业和群众办事便利度、满意率。深化“一门一网一次”改革，在政务服务大厅全面推行“一窗受理、集成服务”模式，让更多事项“一网通办”，必须到现场办的力争做到“只进一扇门”“最多跑一次”。探索开展营商环境自我评价，推动营商环境迈入全国第一方阵。

　　提升行政效能。坚持刀刃向内、自我革命，深入推进政府机构改革，构建权责协同、运行高效的政府治理体系。改进年度目标责任考核机制，进一步激励广大干部担当作为。全面深化、拓展、延伸“13710”信息化督办制度，坚决破除形式主义、官僚主义，坚决整治不担当、不作为、慢作为、假作为，全力打通“中梗阻”，提高执行力。

　　坚守廉政底线。深入推进政府系统党风廉政建设和反腐败斗争，严格执行中央八项规定精神和省、市实施办法。继续深化警示教育，巩固巡视整改成果，推动形成长效机制，加强对重点领域、关键环节、重要岗位权力运行的监督制约。严肃查处违纪违法案件，树立清正廉洁的良好形象。

　　各位代表！实干创造未来，奋斗成就梦想。让我们更加紧密地团结在以习近平同志为核心的党中央周围，认真贯彻省委、省政府各项决策部署，在市委的坚强领导下，迎难而上、真抓实干，推动新时代美丽晋城高质量转型发展取得新成果，以优异成绩迎接中华人民共和国成立70周年！
